# A new immunochromatographic assay for on-site detection of porcine epidemic diarrhea virus based on monoclonal antibodies prepared by using cell surface fluorescence immunosorbent assay

**DOI:** 10.1186/s12917-019-1773-4

**Published:** 2019-01-18

**Authors:** Hongfen Bian, Fei Xu, Yumin Jia, Lei Wang, Shengchao Deng, Aiqing Jia, Yong Tang

**Affiliations:** 1 Department of Bioengineering, Guangdong Province Key Laboratory of Molecular Immunology and Antibody Engineering, Jinan University, Guangzhou, 510632 People’s Republic of China; 2 Guangdong Haid Institute of animal Husbandry & Veterinary, Guangzhou, 511400 People’s Republic of China; 3 College of veterinary medicine, Hunan Agricultural University, Changsha, 410128 People’s Republic of China; 4 Institute of Food Safety and Nutrition, Jinan University, Guangzhou, 510632 People’s Republic of China

**Keywords:** Porcine epidemic diarrhea virus, Cell surface fluorescence immunosorbent assay, Immunoassay, South of China, Swine stool analysis

## Abstract

**Background:**

Porcine epidemic diarrhea virus (PEDV) is a highly effective pathogen that can cause death of new-born piglet, resulting in big economical loss in pig farming industry. For rapid detection of PEDV, a new immunochromatographic assay (ICA) based on monoclonal antibodies (mAbs) was developed in this study.

**Results:**

The mAbs were prepared by using PEDV positive hybridoma cells that were selected by using cell surface fluorescence immunosorbent assay (CSFIA). Fourteen mAbs against PEDV strain isolated from south of China were prepared. The optimal mAb 4A11 was coated on NC membrane as the capturing reagent and the mAb A11H7 was coupled to gold nanoparticles (AuNPs) as detection reagent for the new ICA. The new ICA was used to measure PEDV in phosphate buffer containing tween-20. Results indicated that the limit of detection (LOD) of the new ICA was 0.47 μg/mL (5.9 × 10^3^ TCID_50_/mL) and the liner detection range of the ICA was 0.625–10 μg/mL (7.8 × 10^3^–10^5^ TCID_50_/mL). The specificity analysis results showed that this new ICA had no cross reaction in the presence of other porcine viruses. The ICA was also validated for the detection of PEDV in swine stool samples with little interference from swine stool. To compare its accuracy to other traditional detection methods, 27 swine stool samples from south of China were investigated with the new developed ICA, commercial strip and RT-PCR. Results showed that the new ICA was more comparable to RT-PCR than commercial test strip.

**Conclusions:**

A new ICA based on mAbs prepared by CSFIA was developed in this study. It was a sensitive, specific and rapid method that could be used for on-site detection of PEDV and therefore was useful for the diagnosis and prevention of PED.

**Electronic supplementary material:**

The online version of this article (10.1186/s12917-019-1773-4) contains supplementary material, which is available to authorized users.

## Background

Porcine epidemic diarrhea virus (PEDV), which belongs to the genus Alphacoronavirus and the family Coronaviridae, is an enveloped single-stranded positive-sense RNA virus [1, 2]. PEDV is a highly effective pathogen responsible for causing porcine epidemic diarrhea (PED), a disease characterized by severe and acute watery diarrhea, dehydration and vomiting that results in high mortality rate of one-week-old piglets [3, 4]. Since the first appearance in England in the early 1970s, outbreaks of PED have been reported in several European and Asian countries [5, 6]. PED was first reported in China in 1973, followed by a large-scale outbreak of PED in December 2010 that led to heavy economic loss [7, 8]. Therefore, it is of great importance to properly and routinely monitor PEDV.

Unfortunately, due to the fact that PEDV and a different Alphacoronavirus, transmissible gastroenteritis virus (TGEV), have the same epidemiological and clinical features. These two viruses have led to complications in clinical diagnosis. Therefore, it is of great importance to develop differential laboratory tests [9, 10]. To date, several methods including RT-PCR [11], separation identification [12], serological method [13], enzyme-linked immunosorbent assay (ELISA) [14] and colloidal gold method [15–17] have been used to detect the PEDV antigen. Although RT-PCR serves as a good standard due to its high sensitivity and accuracy, this method relies heavily on sophisticated equipment and expensive apparatus. Several other common methods are also not suitable for on-site diagnosis due to the following reasons. Separation identification means to isolate PEDV in Vero cell cultures from the small intestine of a PEDV infected piglet. It is a classical method for PEDV detection, but it is time-consuming for it needs at least one week. The serological method can only detect PEDV antibody. However, whether the PEDV antibody is from vaccine infection or wild infection is still unknown. ELISA is a cheap and simple approach for PEDV detection, however, it involves long incubation period, multiple washing steps, and the assistance of a microplate reader. In contrast, recently developed immunochromatographic assay (ICA) is much more applicable for on-site diagnosis of PEDV for it is simple, sensitive, specific and can be operated without training. Therefore, this method could be useful for monitoring the infection of PED [18–20].

Currently, the most widely used commercial PEDV test strip in China (BIONOTE test strip), which is made by utilizing ICA, is based on the PEDV-DR13 strain. The strain was harvested from a suspension of minced small intestine from infected neonatal pigs in south of Korea [21]. Considering regional differences in strains, it might not be the best choice for PEDV detection in south of China. A PEDV strain CHYJ130330 was isolated from the fecal specimen of a 3-day-old dead piglet in March 2013 on a commercial swine farm in Guangdong Province in the south of China [22]. In this study, we prepared monoclonal antibodies (mAbs) using cell surface fluorescence immunosorbent assay (CSFIA) [10], a simple and rapid method for selecting positive hybridoma cells. Fourteen mAbs against this PEDV strain were prepared. Relying on signals emitted from gold nanoparticles labeled mAb (AuNPs-mAb), a new ICA was developed for sensitive, specific and on-site detection of PEDV in swine stool in China. A comparison between RT-PCR, BIONOTE test strip and the ICA was performed to confirm the applicability of the newly developed ICA for on-site PEDV detection.

## Methods

### Materials and reagents

Vero cell, porcine epidemic diarrhea virus (PEDV) strain named CHYJ130330, pseudorabies virus (PRV), classical swine fever virus (CSFV), transmissible gastroenteritis virus (TGEV) and swine stool were obtained from Guangdong Haid Institute of Animal Husbandry & Veterinary (Guangdong, China). Porcine reproductive and respiratory syndrome virus (PRRSV) and porcine circovirus-2 (PCV-2) were obtained from Winsun Bio (Guangdong, China). Balb/c mice were purchased from Southern Medical University. Myeloma cells (SP2/0) were purchased from Shanghai Cell Biology (Shanghai, China). GibicoRPMI-1640 was purchased from Asegene (Guangzhou, China). Ninety-six-well polystyrene plates were purchased from JET BIOFIL (Guangzhou, China). Goat anti-mouse IgG H&L (HRP) was purchased from Abcam (Cambridge, MA). MAb subtype classification kit was purchased from sigma (USA). The HRP-labeling kit was purchased from GalaxyBio (Beijing, China). BCA kit was purchased from Thermo (USA). Nitrocellulose (NC) membrane (HF18002S25), conjugate pad, sample pad, plastic backing and absorbent pad were purchased from Millipore (Shanghai, China). PEDV Ag test kit was obtained from BIONOTE (Korea). Ultrapure water produced by a Milli-Q Ultra Pure System (Millipore, USA) was used throughout this study. All chemicals used were of analytical grade or higher.

### Apparatus

Absorbance of the HRP-based ELISA was measured using a Synergy H1 Hybrid Multi-Mode microplate reader (Bio-Tek Instruments, Inc., Winooski, VT). The following equipments were also utilized in this study: centrifuge 5810 R (Eppendort, Germany), XW-80 Avortex Mixers (Shanghai, China), magnetic stirrer (Beike, Beijing), dynabeads MX mixer (Invitrogen, America), programmable strip cutter HGS201 (AUTOKUN, China), XYZ3060 platform (Bio-Dot Scientific Equipment, China), and electrothermal forced air convection drying oven (TAISITE Instrument, China).

### Preparation of PEDV

PEDV was inoculated onto a Vero cell monolayer for 45 min at 37 °C. Cell infection at a MOI of 0.005. Then maintenance medium was added and cultured for 2 or 3 days. When the cytopathic changes reached above 85%, the cells were collected and repeatedly frozen and thawed for 3 times. After ultrasonic cracking and centrifugation at 10000 rpm for 1 h, the supernatant was further centrifuged at 45000 rpm for 3 h and the precipitate was resuspended in PBS (0.01 M, pH 7.4). The crude extraction virus was successively added into 30%~ 60% sucrose-PBS and centrifuged at 33000 rpm for 3 h. Then it was resuspended in PBS and centrifuged at 40000 rpm for 3 h to remove sucrose content. The purified virus was resuspended in PBS. The uninoculated Vero cells were subjected to the same treatment and used as negative control. The pre-purification protein concentration was measured by Nanodrop2000 micro nucleic acid tester. Virus titer was expressed as TCID_50_, 10^6^ TCID_50_/mL.

### Preparation and purification of monoclonal antibody against PEDV

**Fig. 1:**
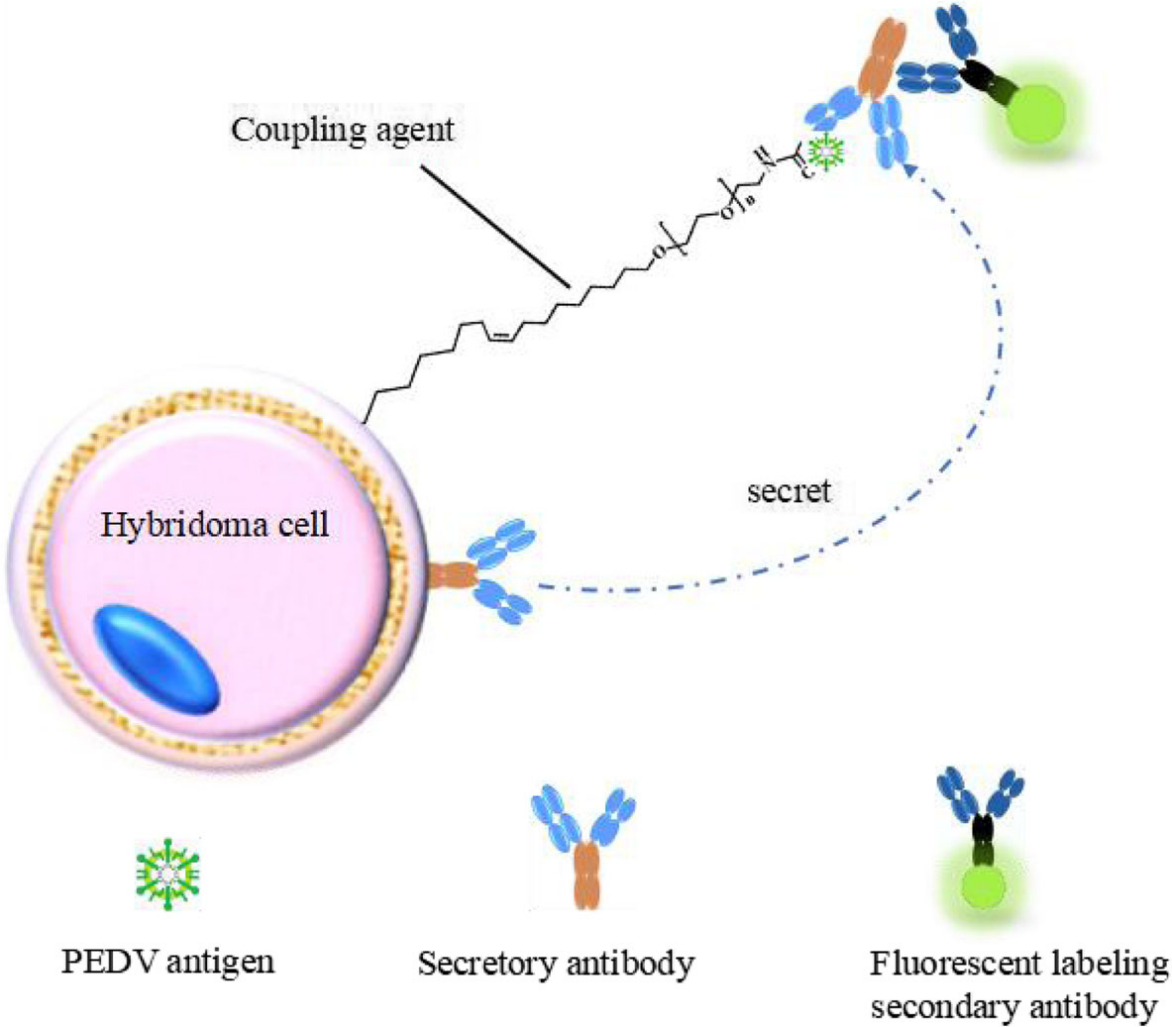
The mechanism of CSFIA based on the specific binding of antigens anchored on the positive hybridoma cell surface and capturing of secreted antibodies from the same cells

Viral protein concentration was measured using BCA, 80 μg/mL. 1.25 mL of the purified PEDV antigen and an equal volume of Freund’s complete adjuvant, was injected into each mouse (6 weeks, female, Balb/c) for the first immunization. Mice were immunized every 2 weeks. Tail blood was taken on the tenth day after the third immunization. Mice with high titer were selected for fusion 3 days after they were boosted with immune antigen PEDV. To prepare the hybridomas, the mice were euthanized by cervical dislocation. All the mice were sent to Huaqiao Hospital for centralized treatment after the study. CSFIA, a simple and rapid method for selecting positive hybridoma cells, was used to screen for positive hybridoma cell clones. In this study, PEDV antigens were first anchored to the hybridoma cell surface through a dual functioning molecular Oleyl-PEG4000-NHS. Specific antibodies secreted from hybridoma cells were then captured by the antigens on the cell surface. Positive hybridoma cells were stained using a fluorescently labeled anti-mouse IgG-Fc antibody (Fig. 1). After the addition of a methylcellulose semisolid medium, positive clones were picked using a pipet. These positive cell clones were used to produce monoclonal antibodies after direct expansion [10].

MAbs were prepared by inducing ascites in mice. They were then purified by ammonium sulphate, desalt column, and protein G affinity chromatography column. MAbs were preserved at − 20 °C after freeze-drying. Titer of mAbs was determined by indirect ELISA with PEDV as coating antigen and HRP-goat anti-mouse IgG as secondary antibody. The sensitivity of mAbs was determined by indirect competition ELISA with PEDV as coating antigen and PEDV specific HRP-monoclonal antibody as the competitors. The mAb classification kit was used to identify the category and subclass of immunoglubulin. The mAb pair for PEDV detection was selected by double antibody sandwich ELISA matrix method with HRP labeled mAbs as secondary antibodies and mAbs as coating substrates.

### Preparation and optimization of the new ICA

#### The preparation of gold nanoparticles (AuNPs) and gold nanoparticles labeled mAb (AuNPs-mAb)

To prepare AuNPs, trisodium citrate dehydrate was used to reduce gold chloride (HAuCl_4_). In brief, 50 mL ultrapure water was added to a clean triangle flask and heated and stirred on a magnetic stirrer until boil. Then, 1 mL of 1% HAuCl_4_ was quickly added to the triangle flask and 1.2 mL trisodium citrate dihydrate (10 mg/mL) was added after a few seconds. The solution was heated for 10 min, and then moved to an unheated plate for continued stirring. The prepared AuNPs were then diluted with ultrapure water to 50 mL.

AuNPs-mAb was prepared by labeling mAb to AuNPs. First, 1 mL of AuNPs (0.02 mg/mL) was added to a clean centrifuge tube, and 16 μL of 0.25 M K_2_CO_3_ were added; the solution was mixed on an Avortex Mixers. Then, 2 μL mAb was quickly added to the centrifuge tube with mixing. The mixture was rotated for 15 min and then kept still for 15 min at room temperature. Subsequently, 100 μL of 10% BSA were added to cover the unconjugated site. The mixture was rotated for another 15 min and then kept still for 15 min at room temperature. Finally, the mixture was centrifuged at 11000 rcf for 15 min and the precipitate was resuspended in 60 μL PBS (0.015 M, pH 7.4, containing 20% (*w*/*v*) sucrose, 20% (w/v) trehalose, 0.1% (w/v) PVP K40, 0.1% (w/v) S-9, 1% (w/v) BSA, and 0.5% (w/v) Tween-20).

### Assembly of the new ICA

**Fig. 2:**
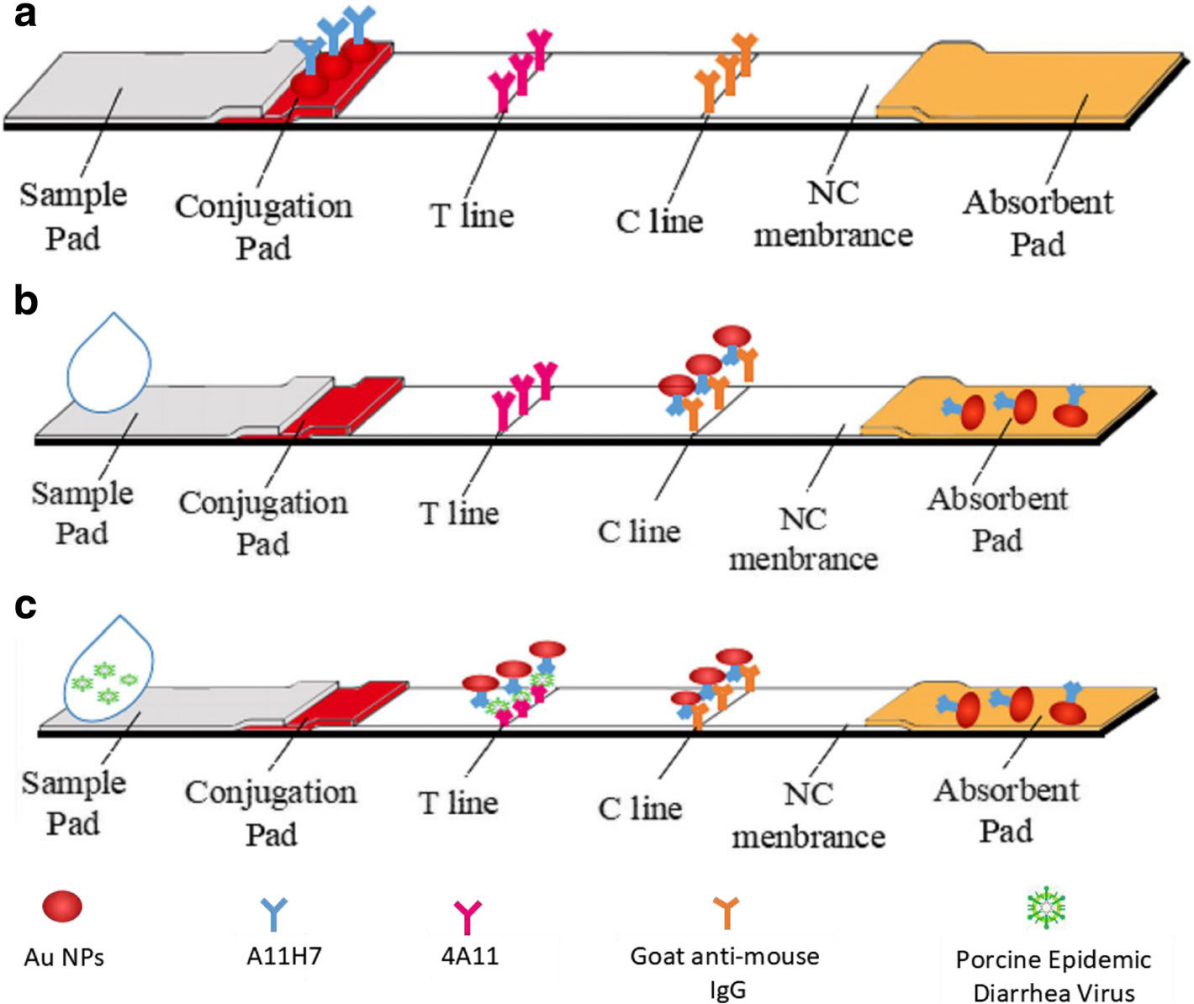
The simple schematic diagram of the new ICA. **a**. The stereoscopic structure of the new ICA; **b**. Negative reaction. When negative spiked samples were added to the sample pad, no red line on the T-line was observed; **c**. Positive reaction. When positive spiked samples were applied to the sample pad, a red line was visible on the T-line

This new ICA was sandwich type, in which two monoclonal antibodies were used separately as capture and detecting reagents to detect PEDV (Fig. 2). Gray value corresponding to signal from the red T-line was obtained using image J [23–25]. The assembly of the new ICA was as follows: The new ICA was made of PVC plate, NC membrane, sample pad, conjugate pad and absorbent pad. PBS (0.015 M, pH 7.4) diluted mAb (0.875 mg/mL) and goat anti-mouse IgG (1 mg/mL) were dispensed on specific areas of NC membrane called the test line (T-line) and calibration line (C-line) using an automatic dispenser at a volume of 1 μL/cm. The NC membrane was dried at 37 °C and saved for later use. The AuNPs-mAb was loaded onto the conjugate pad using an automatic dispense system set to 5.0 μL/cm and then dried at 37 °C. The pretreatment of sample pad involved soaking in PBS (0.015 M, pH 7.4, containing 2.5% (*w*/*v*) sucrose, 2% (w/v) BSA, and 1% (w/v) Tween-20) and drying at room temperature and then at 37 °C. The PVC plate acted as the holder of the new ICA, onto which sample pad, conjugate pad, NC membrane and absorbent pad were pasted with an overlap of 2 mm between each part. After the assembly was completed, the new ICA was cut into 3.8-mm-wide and 60-mm-long strips using a programmable HGS201 strip cutter and kept in a suitable plastic cassette for further use. A fully assembled new ICA is shown in Fig. 2a.

### Optimization of the new ICA

To optimize the quality of the new ICA, some important single factors were optimized. They were capture and detection mAb, the size of gold nanoparticles, the type of sample pad, the type of conjugate pad, the type of Nitrocellulose membrane, the type of absorbent pad, the amount of tween-20 addition and the spray volume of AuNPs-mAb. The optimization methods are shown in the supplemental materials.

### Performance of the new ICA for PEDV detection in PBT

#### Sensitivity of the new ICA

To determine the sensitivity of the new ICA, the concentrated PEDV was diluted into a series of concentrations using PB (0.2 M, pH 7.4, containing 1% (*w*/*v*) Tween-20) as sample solutions. Then, 80 μL of different sample solutions were added to the sample holes of the new ICAs respectively. Photos were taken to record the results after 15 min of reaction.

#### Specificity of the new ICA

To verify the specificity of the new ICA, PEDV, PRV, PCV-2, PRRSV, CSFV, and TGEV at the same concentrations were prepared and tested following similar procedures.

#### Stability of the new ICA

The stability of the new ICA was tested by comparing results from the experiments performed now to those from experiments performed a month later using the same ICAs.

#### Detection of PEDV in spiked samples

For the detection of PEDV in spiked samples, negative samples with different PEDV concentrations were prepared. First, 800 μL PB (0.2 M, pH 7.4, containing 1% (*w*/*v*) Tween-20) was added to a 1.5 mL centrifuge tube. Then, 200 μL negative swine stool A7 was added to the solution and mixed for 5 min. After centrifugation at 11000 rcf for 15 min, the solution was kept still for 15 min. The supernatant was collected and the precipitate was discarded. PEDV was incorporated into the sample at 0.1, 1, 5, 10 and 20 μg/mL. Finally, 80 μL of the spiked solutions were added to the sample holes of the new ICAs respectively. Photos were taken to record the results after 15 min of reaction.

#### Detection of PEDV in swine stool samples

For detection of PEDV in swine stool samples, 50 μL of swine stool samples and 50 μL of PB (0.2 M, pH 7.4, containing 1% (*w*/*v*) Tween-20) were mixed and incubated for 5 min. After centrifugation at 11000 rcf for 15 min, the solution was kept still for 15 min. The supernatant was collected and the precipitate was discarded. The swine stool samples were analyzed using RT-PCR, BIONOTE test strips and the new ICA, respectively.

## Results

### Preparation and characterization of antibody to PEDV

Cell lesion was above 85% after PEDV was inoculated onto Vero cell monolayer for 36 h. After repeated freezing and thawing, cracking and sucrose-PBS gradient centrifugation, the virus bands between the 40 and 60% interface were collected. After identification, protein concentration was determined to be 4 mg/mL. The prepared PEDV was used to immunize mice to generate mAbs against PEDV [24]. From the hybridoma cells generated against PEDV, 14 clones that could produce mAbs with strong PEDV binding ability were selected. Immunoglobulin isotyping suggested these clones were IgG2a. To select PEDV mAb pairs with high sensitivity and specificity, a double antibody sandwich ELISA was used. The amount of capture antibody for coating and detecting antibody in both experimental and control group was 10 μg/ml. 1 μg/ml PEDV was added to each well of experimental group while an equal volume of PBS was added to the control group. Only the mAb pairs which have strong signal (OD ≥ 1.0) in PEDV group and no obvious signal (OD ≤ 0.2) in PBS group were marked as positive. All the mAb pairs which have no strong signal in PEDV group or have obvious signal in PBS group were marked as negative. As shown in Tables 1, 5 of the 14 mAbs were successfully paired using double antibody sandwich ELISA matrix method. These successfully paired mAbs (4A11, 5H9, 5A9, A11H7 and 4H7) were used in the new ICA for their outstanding binding ability.

**Table 1:** The selection results of paired antibodies in double antibody sandwich ELISA

		Capture antibody													
		A11H7	5H12	4D5	4A11	5E2	2C11	2H2	4H7	3G10	5H9	5A9	3G9	3C11F5	3C11F9
Detection antibody	HRP-A11H7	-	-	-	++	-	-	-	-	-	-	-	-	-	-
	HRP-5H12	-	-	-	-	-	-	-	-	-	-	-	-	-	-
	HRP-4D5	-	-	-	-	-	-	-	-	-	-	-	-	-	-
	HRP-4A11	-	-	-	++	-	-	-	-	-	-	-	-	-	-
	HRP-5E2	-	-	-	-	-	-	-	-	-	-	-	-	-	-
	HRP-2C11	-	-	-	-	-	-	-	-	-	-	-	-	-	-
	HRP-2H2	-	-	-	-	-	-	-	-	-	-	-	-	-	-
	HRP-4H7	-	-	-	-	-	-	-	-	-	-	++	-	-	-
	HRP-3G10	-	-	-	-	-	-	-	-	-	-	-	-	-	-
	HRP-5H9	-	-	-	-	-	-	-	-	-	-	-	-	-	-
	HRP-5A9	-	-	-	-	-	-	-	-	-	++	-	-	-	-
	HRP-3G9	-	-	-	-	-	-	-	-	-	-	-	-	-	-
	HRP-3C11F5	-	-	-	-	-	-	-	-	-	-	-	-	-	-
	HRP-3C11F9	-	-	-	-	-	-	-	-	-	-	-	-	-	-

### Parameter optimization of the new ICA

First, the new ICA was optimized by selecting the best pair from the 5 selected mAbs. The optimization results showed that 4A11 was the best capturing mAb and A11H7 was the best detection mAb (see Additiona file 1: Figure S1. in the supporting information, number 10 represent 4A11 as capturing mAb and A11H7 as detection mAb).

In addition, since gold nanoparticles of suitable size is essential to a successful ICA, an assay was performed to optimize the size of gold nanoparticles. In brief, 16 nm, 24 nm, 30 nm and 40 nm AuNPs were used to label mAb A11H7. With increasing size of gold nanoparticles, the red signal of the T-line also increased, while the red signal of the C-line remained the same (see Additional file 2: Figure S2. in the supporting information). In addition, the reaction was not blocked at any time during the process despite the increasing size of the gold nanoparticles. We found that AuNPs of 40 nm was associated with the strongest red signal at the T-line with to no impairment to the reaction process. Therefore, 40 nm was chosen as the optimized size for AuNPs for subsequent experiments (see Additional file 2: Figure S2. in the supporting information).

Interestingly, the type of sample pad, conjugate pad, NC membrane and absorbent pad also appeared to have great impact on reaction rate of the new ICA. To optimize these factors of the ICA, different types of sample pad, conjugate pad, NC membrane and absorbent pad were tested in the optimization experiment. As shown in Additional file 3: Figure S3. in the supporting materials, the new ICA with GL-B04 as sample pad showed the most uniform red C line without any break. Therefore, GL-B04 was chosen as the optimized sample pad. It can be seen from Additional file 4: Figure S4. in the supporting materials, the new ICA with polyester film as conjugate pad showed the most uniform red C line without any break. Thus, polyester film was selected as the optimized conjugate pad. What can be learned from Additional file 5: Figure S5. was that the new ICA with MILLIPORE 135 as NC membrane showed the most uniform red C line without any break. Therefore, MILLIPORE 135 was chosen as the optimized NC membrane. As for the optimization of absorbent pad, the new ICA with H2 as absorbent pad (see Additional file 6: Figure S6. in the supporting materials) showed strong color signal at the T-line. Therefore, H2 was chosen as the optimal absorbent pad for the new ICA.

As tween-20 can remove unbound AuNPs-mAb on the T-line and reduce nonspecific binding. Sample solutions with different volumes of tween-20 (5, 10, 15, 20 and 40 μL/mL) were added to ICAs. Results confirmed that no red line on the T-line was observed in the negative sample. At 15 μL/mL upward, stronger red color at the T line of negative sample was observed with increasing volume of tween-20 added (see Additional file 7: Figure S7. in supporting information). Results for positive sample remained almost the same with increasing volume of tween-20. Therefore, 10 μL of tween-20 to 1 mL of sample solution was chosen as the optimized ratio for subsequent experiments.

Furthermore, the spray volume of AuNPs-mAb was also important to the new ICA. As shown in Additional file 8: Figure S8. in supporting information, no red line at the T-line of the negative sample ICA was observed until the spray volume of AuNPs-mAb was at 6 μL/cm and the red color on the T-line of the negative sample ICA was more intense with increasing spray volume. However, the red line at the T-line of positive sample test strip remained almost the same. Taking nonspecific adsorption and sensitivity into consideration, 5 μL/cm was chosen as the optimal spray volume for subsequent experiments.

### Performance of the new ICA for PEDV detection in PBT

#### Sensitivity of the new ICA

**Fig. 3:**
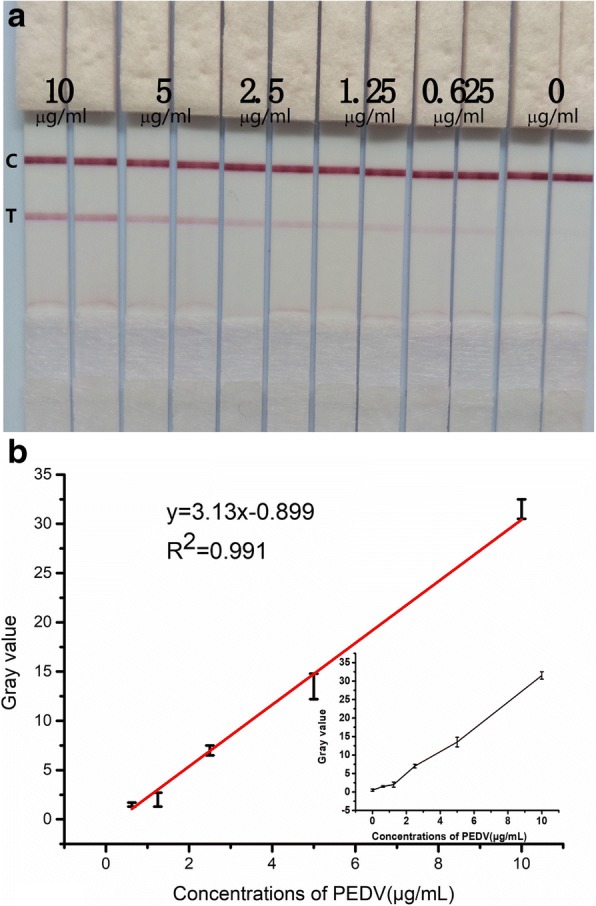
**a**. The new ICA at different PEDV concentrations. Results photographed by a mobile phone. Red color on T-line became more intense with increasing PEDV concentration. **b**. The standard curve plotted using the gray values of the T-line signals obtained by Image J. Each figure represents the mean of 6 replicates

The new ICA was designed for efficient detection of PEDV. The performance of the new ICA for detecting PEDV at various concentrations was tested using a serial dilution of PEDV in PBT (0, 0.625, 1.25, 2.5, 5 and 10 μg/mL) under the optimized condition (Fig. 3a). The limit of detection (LOD) was found to be 0.47 μg/mL (5.9 × 10^3^ TCID_50_/mL).

To quantitatively analyze the performance of the new ICA, gray values obtained from Image J were used to draw a standard curve in origin 7.0 (Fig. 3b). The liner detection range was 0.625 μg/mL to 10 μg/mL (7.8 × 10^3^ to 10^5^ TCID_50_/mL) and the coefficient (R^2^) was 0.991. The exact value of PEDV concentration could be read directly by comparing results and the photos corresponding to the standard curve. These results indicated that this new ICA could determine PEDV in a precise and quantitative manner.

#### Specificity of the new ICA

**Fig. 4:**
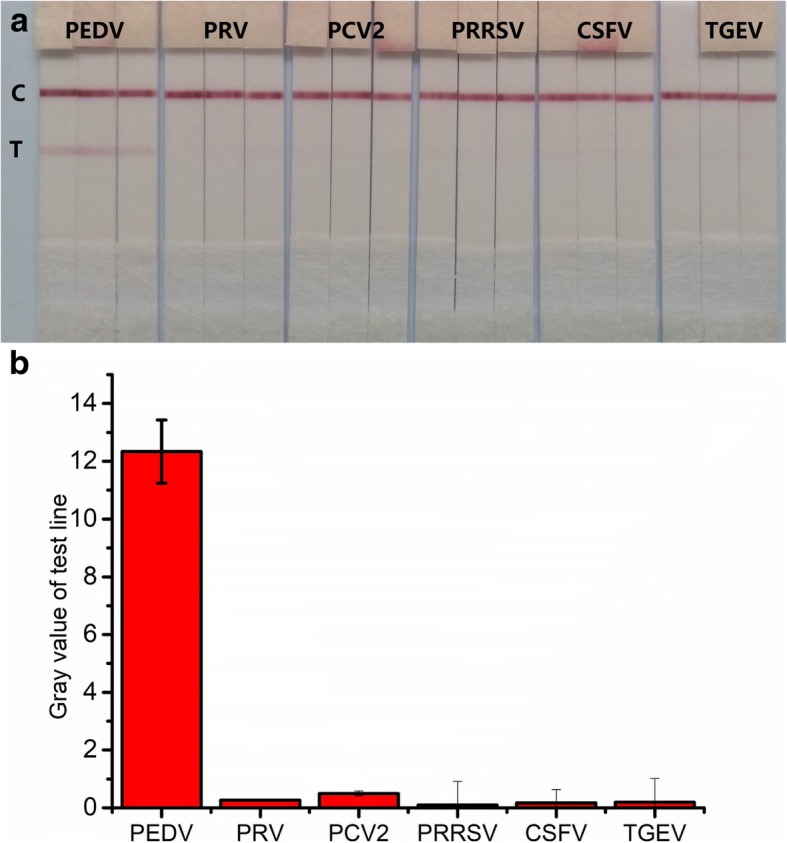
Specificity of the new ICA. **a**. Image of the new ICA for detection of different swine viruses. **b**. Cross-reaction of the new ICA with other swine viruses. Each figure represents the mean of 6 replicates

To confirm the specificity of the new ICA, 5 kinds of swine viruses were tested and PEDV was used as positive control. These swine viruses were assayed by the new ICA at the same concentrations as PEDV. The red line at the T-line was obvious in PEDV sample, while it was invisible in samples containing other swine viruses (Fig. 4a). These results indicated high specificity of the new ICA. Results were further analyzed using gradation histogram made by origin 7.0 based on gray values obtained from Image J. No significant cross-reaction of other swine viruses was observed in the developed new ICA (Fig. 4b), which further confirmed its high specificity.

#### Stability of the new ICA

To verify the stability of the new ICA, experiments were carried out at different time points using the same ICA and PEDV. In both experiments, the positive sample solution showed a red line at T-line while the negative sample solution did not (see Additional file 9: Figure S9. in the supporting information). These results indicated that the new ICA was stable over time.

#### Detection of PEDV in spiked samples

Negative swine stool was mixed with PEDV to prepare the spiked samples in this study. Spiked samples that included 0.1, 1, 5, 10, or 20 μl PEDV were tested. The red color at the T-line became more intense with increasing PEDV concentration. Each spiked sample was analyzed in six repeated experiments and the gray values were calculated using the standard equation corresponding to the standard curve. As shown in Table 2, the recoveries of the new ICA ranged from 90 to 110.27% with the highest relative standard deviation (RSD) calculated to be 12.63%. The result indicated that the new ICA was ideal for detection of PEDV in spiked samples.

**Table 2:** Detection results of PEDV in spiked samples

Added (μg/ml)	Found (μg/ml)	Recovery(%)	RSD(%)
0.1	0.09 ± 0.002	90	2.22
1	0.99 ± 0.09	99.75	9.02
5	4.75 ± 0.6	95	12.63
10	11.03 ± 0.8	110.27	7.25
20	19.92 ± 2.4	99.6	12.05

Notes: Results were expressed as mean ± S.D. (*n* = 6). Recovery (%) = (Found/Added) × 100%. RSD = (S.D./mean) × 100%

#### Detection of PEDV in swine stool samples

To analyze the performance of the new ICA in detecting PEDV in swine stool samples, 29 swine stool samples were collected from different swine farms from south of China. RT-PCR, commercial BIONOTE test strip and the new ICA were used to test these swine stool samples, respectively. As shown in Table 3, the total coincidence of BIONOTE test strip to RT-PCR was 62.69% while that to the new ICA was 74.07%, indicating that the new ICA was more accurate than BIONOTE test strip.

**Table 3:** Detection results of PEDV in swine stool samples from south of China

Farm name	Sample quantity	Positive detection result			Co-positive detection result		Co-negative detection result		Coincidence rate with RT-PCR	
		RT-PCR	BIONOTE test strip	Lab test strip	BIONOTE test strip	Lab test strip	BIONOTE test strip	Lab test strip	BIONOTE test strip	Lab test strip
Farm 1	7	5	3	7	3	5	2	0	71.43%	71.43%
Farm 2	6	5	2	4	2	4	1	1	50%	83.33%
Farm 3	8	6	1	7	1	5	2	0	37.50%	62.50%
Farm 4	4	4	4	3	4	3	0	0	100%	75%
Farm 5	2	2	2	2	2	2	0	0	100%	100%
Total	27	22	12	23	12	19	5	1	62.69%	74.07%

## Discussions

In the present study, an immunochromatographic assay for PEDV detection was developed and tested with spiked samples and swim stool samples from field infected pigs. The assay was based on mAbs to CHYJ130330, which is a new strain of PEDV found in south of China and therefore is an appropriate target antigen for PEDV detection.

First, to prepare PEDV specific monoclonal antibody, immune antigen PEDV was cultured and purified in the normal way to ensure the quality of immune effect. T he well prepared 14 PEDV mAbs were used to do a double antibody sandwich ELISA matrix, in which these 14 mAbs were used as capture antibody and the same 14 mAbs labeled with HRP were used as detection antibody. The aim was to find which mAb as capture antibody and which HRP-mAb as detection antibody can produce the best detection result. That means, which mAb pair had the best binding ability to PEDV. Results showed that 4A11, 5H9, 5A9, A11H7 and 4H7 were these mAbs that can pair successfully.

Second, due to the fact that the paired antibodies selected by double antibody sandwich ELISA matrix may not be suitable for the new ICA due to the differences in materials and environment, the new ICA was optimized by selecting the best pair from the 5 selected mAbs. 4A11 was proved to be the best capturing mAb while A11H7 was the best detection mAb. This optimization experiment greatly improved the sensitivity of the new ICA.

Third, gold nanoparticles were important for a successful ICA. If they were too big, they would block NC membrane and weaken red signal of T and C line. On the other hand, if too small, the red signal of T and C line was weak and it was not easy to observe it. Considering both, the AuNPs of 40 nm were found to be the optimal AuNPs in this study. The spray volume of AuNPs-mAb was also optimized. If the spray volume was too high, it was easy to produce nonspecific adsorption. However, if it was too low, the result would have low sensitivity. 5 μL/cm was selected considering both sides.

Forth, different types of sample pad, conjugate pad, NC membrane and absorbent pad had different influences on running speed, releasing amount and nonspecific absorption. So they were optimized. Results showed that the optimized condition was GL-B04 as sample pad, polyester film as conjugate pad, MILLIPORE 135 as NC membrane and H2 as absorbent pad.

Fifth, tween-20 in sample solution acted as surface active agent, which can accelerate running speed. A high running speed may be not good because it can reduce sensitivity. Therefore, the tween-20 concentration was optimized and results showed that 10 μL of tween-20 added to 1 mL of sample solution was the best choice.

Specific experiment showed that there was no cross reaction between this ICA and other swine viruses, indicating that this ICA can distinguish PEDV from other swine viruses. When detecting PEDV in PBT, the LOD was 0.47 μg/mL (5.9 × 10^3^ TCID_50_/mL) and the liner detection range was 0.625–10 μg/mL (7.8 × 10^3^ -10^5^ TCID_50_/mL). The PEDV concentration in cultured PEDV was pretty high. Therefore, it can be used to detect cultured PEDV. The data obtained by the new ICA were compared with those obtained by both RT-PCR and commercial strip. It has been described that RT-PCR is the most sensitive and trustworthy method for PEDV detection. In this study, the commercial strip was included for comparison purpose. The data analyzed in the present work showed that the total coincidence of BIONOTE test strip to RT-PCR was 62.3% while that to the new ICA was 74.07%, indicating that the new ICA was more comparable to RT-PCR than commercial test strip. These results suggested that the new ICA was suitable for monitoring PEDV.

The new ICS shows pretty good detection ability of detecting PEDV in swine stools, which will help pig farms in diagnosis of PEDV. Based on the success of making this new ICS, double pathogen detection is going to be done. It can satisfy the need of pig farm. In addition, PEDV fluorescent test strips will be done for precise quantitative detection. We believe that these new ICAs will bring great help to agriculture.

## Conclusions

In conclusion, a new ICA based on mAbs prepared by CSFIA was developed in this study. The gold nanoparticle signal of the test strip was of superior strength with very low background noise. The LOD of the new ICA was 0.47 μg/mL (5.9 × 10^3^ TCID_50_/mL) and the liner detection range was 0.625 to 10 μg/mL (7.8 × 10^3^ to 10^5^ TCID_50_/mL). In addition, the new ICA exhibited high specificity with little interference from swine stool, and could achieve recoveries ranging from 90 to 110.27% in spiked samples. Compared to RT-PCR and ELISA, the new ICA does not require sophisticated instruments and training. Relative to commercial test strips, the new ICA provides more accurate detection of PEDV in swine stool samples from south of China. Our study highlighted the potential of the on-site application of this new ICA for diagnosis and prevention of PED.

## Additional files

Additional file 1: **Figure S1.** Selection of capture and detection of mAbs of the new ICA for PEDV detection. (DOC 135 kb)

Additional file 2: **Figure S2.** Optimization of the size of gold nanoparticles. Each point was photographed with two copies. (DOC 112 kb)

Additional file 3: **Figure S3.** Optimization of the type of sample pad. (DOC 376 kb)

Additional file 4: **Figure S4.** Optimization of the type of conjugate pad. (DOC 396 kb)

Additional file 5: **Figure S5.** Optimization of the type of NC membrane. (DOC 413 kb)

Additional file 6: **Figure S6.** Optimization of the type of absorbent pad. Each point was photographed with two copies. (DOC 328 kb)

Additional file 7: **Figure S7.** Optimization of the amount of tween-20 addition. Each point was photographed with two copies. (DOC 609 kb)

Additional file 8: **Figure S8.** Optimization of the spray volume of AuNPs-mAb. (DOC 124 kb)

Additional file 9: **Figure S9.** Stability of the sandwich ICA for PEDV detection. a There was red line on the positive test strip’s T line. b There was red line on the positive test strip’s T line when the same experiment was done with the same test strips made one month ago. (DOC 163 kb)

## Abbreviations

CSFIA: Cell surface fluorescence immunosorbent assay
CSFV: Classical swine fever virus
ICA: Immunochromatographic assay
mAbs: Monoclonal antibodies
PCV-2: Porcine circovirus-2
PEDV: Porcine epidemic diarrhea virus
PRRSV: Porcine reproductive and respiratory syndrome virus
PRV: Pseudorabies virus
TGEV: Transmissible gastroenteritis virus

## References

[1] DA B RSB. Coronavirus genome structure and replication. Current TopICA in Microbiology and Immunology. 2005:1–30. 10.1007/3-540-26765-4_1 PMC7120446 15609507

[2] A B, R K, K T, A C, M. A (1998) Further analysis of the genome of porcine epidemic diarrhoea virus. Adv Exp Med Biol 781–786. 10.1007/978-1-4615-5331-1_101 9782358

[3] D S, B P (2012) Porcine epidemic diarrhoea virus: a comprehensive review of molecular epidemiology, diagnosis, and vaccines. Virus Genes 2:167–2175. 10.1007/s11262-012-0713-1 PMC7089188 22270324

[4] Pijpers A, van Nieuwstadt A, Terpstra C, Verheijden J (1993). Porcine epidemic diarrhoea virus as a cause of persistent diarrhoea in a herd of breeding and finishing pigs. Vet Rec.

[5] Wood E (1977). An apparently new syndrome of porcine epidemic diarrhoea. Vet Rec.

[6] Jung K, Saif LJ (2015). Porcine epidemic diarrhea virus infection: etiology, epidemiology, pathogenesis and immunoprophylaxis. Vet J.

[7] Bi J, Zeng S, Xiao S (2012). Complete genome sequence of porcine epidemic diarrhea virus strain AJ1102 isolated from a suckling piglet with acute diarrhea in China[J]. J Virol.

[8] Li W, Li H, Liu Y (2011). New variants of porcine epidemic diarrhea virus. China Emerging infectious diseases.

[9] 1 DSS, 1 BKK, 3 JSO, 3 GWH, 2 JSY, 2 HJM, et al (2006) Multiplex reverse transcription-PCR for rapid differential detection of porcine epidemic diarrhea virus, transmissible gastroenteritis virus, and porcine group A rotavirus. J Vet Diagn Investig 3:278–281. 10.1177/104063870601800309 16789718

[10] Li X, Bian H, Yu S, Xiao W, Shen J, Lan C (2018). A rapid method for antigen-specific Hybridoma clone isolation. Anal Chem.

[11] K I HS, T O SS (1997). Direct and rapid detection of porcine epidemic diarrhea virus by RT-PCR. J Virol Methods.

[12] K K, H K, T K, T N, Y I, T S (1992). Isolation and serial propagation of porcine epidemic diarrhea virus in cell cultures and partial characterization of the isolate. J Vet Med Sci.

[13] DG1 D, S2 L, F2 O, A2 S, T2 C, MH2 F, et al. Porcine epidemic diarrhea virus: an overview of current virological and serological diagnostic methods. Virus Res. 2016:60–70. 10.1016/j.virusres.2016.05.013 PMC7172987 27189041

[14] Lrvc R, Valíček L, Šmíd B, Nevoránková Z (2005). An ELISA optimized for porcine epidemic diarrhoea virus detection in faeces. Vet Microbiol.

[15] KS L, M Y, J K, D K, G H, W N (2017). Development of rapid immunochromatographic strip test for the detection of porcine epidemic diarrhoea virus. Vet Rec.

[16] FX1 W, DY2 Y, YN3 J, L3 H, ZY2 S, Q2 H, et al. Reverse transcription cross-priming amplification-nucleic acid test strip for rapid detection of porcine epidemic diarrhea virus. Sci Rep. 2016:24702. 10.1038/srep24702 PMC4835727 27090105

[17] Kim YK, Lim S-I, Cho I-S, Cheong K-M, Lee E-J, Lee S-O, et al. A novel diagnostic approach to detecting porcine epidemic diarrhea virus: the lateral immunochromatography assay. J Virol Methods. 2015:4–8. 10.1016/j.jviromet.2015.08.024 PMC7119843 26342906

[18] Rivas L, Adl E-M, Serrano L, Altet L, Francino O, Sánchez A (2015). Triple lines gold nanoparticle-based lateral flow assay for enhanced and simultaneous detection of Leishmania DNA and endogenous control. Nano Res.

[19] Man Y, Lv X, Iqbal J, Peng G, Song D, Zhang C (2015). Microchip based and immunochromatographic strip assays for the visual detection of interleukin-6 and of tumor necrosis factor alpha using gold nanoparticles as labels. Microchim Acta.

[20] Nardo FD, Baggiani C, Giovannoli C, Spano G, Anfossi L (2017). Multicolor immunochromatographic strip test based on gold nanoparticles for the determination of aflatoxin B1 and fumonisins. Microchim Acta.

[21] Song DS, Oh JS, Kang BK (2005). Fecal shedding of a highly cell-culture-adapted porcine epidemic diarrhea virus after oral inoculation in pigs [J]. Journal of Swine Health and Production.

[22] Jia A, Feng X, Liu Q (2014). Complete genome sequence of CHYJ130330, a highly virulent strain of porcine epidemic diarrhea virus in South China[J]. Genome announcements.

[23] H S, F X, M X, Q F, Z C, S Z (2017). A new lateral-flow immunochromatographic strip combined with quantum dot nanobeads and gold nanoflowers for rapid detection of tetrodotoxin. Analyst.

[24] Xiao M, Fu Q, Shen H, Chen Y, Xiao W, Yan D, et al. A turn-on competitive immunochromatographic strips integrated with quantum dots and gold nano-stars for cadmium ion detection. Talanta. 2018:644–9. 10.1016/j.talanta.2017.10.002 29136875

[25] Wang W, Liu L, Song S, Xu L, Kuang H, Zhu J (2017). Identification and quantification of eight Listeria monocytogene serotypes from Listeria spp. using a gold nanoparticle-based lateral flow assay. Microchim Acta.

