# Supplementary material for: A new immunochromatographic assay for on-site detection of porcine epidemic diarrhea virus based on monoclonal antibodies prepared by using cell surface fluorescence immunosorbent assay
Source: BMC Vet Res. 2019 Jan 18;15:32. doi: 10.1186/s12917-019-1773-4 (PMC6339306; doi:10.1186/s12917-019-1773-4)
Supplement: Supplementary file 1 — Figure S1. Selection of capture and detection of mAbs of the new ICA for PEDV detection. (DOC 135 kb) [file 12917_2019_1773_MOESM1_ESM.doc]

Selection of capture and detection of mAb for the sandwich ICA

The selection of capture and detection of mAb were analyzed by using an orthogonal experiment with five mAbs (4A11, 5H9, 5A9, A11H7 and 4H7), in which these mAbs were used to label AuNPs and disperse on T line. First, the test strips that were made of different mAbs were coded to 1 to 25. Then 2 μl PEDV (Virus titer was expressed as TCID_50_, 10^6^ TCID_50_/mL; Viral protein concentration was measured using BCA, 80 μg/mL) was diluted in 80 μl of PB (0.2 M, pH 7.4, containing 1% (w/v) Tween-20). Finally, 80 μl of different sample solutions were added to the sample holes of 25 test strips respectively and the photos of these test strips were taken to reserve the results after reaction for 15 min.

**Results**


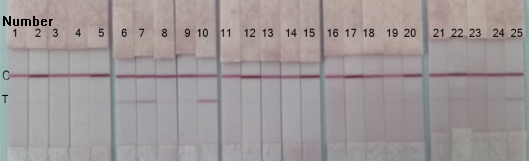


Fig. S1 Selection of capture and detection of mAbs of the new ICA for PEDV detection.
