# Supplementary material for: A new immunochromatographic assay for on-site detection of porcine epidemic diarrhea virus based on monoclonal antibodies prepared by using cell surface fluorescence immunosorbent assay
Source: BMC Vet Res. 2019 Jan 18;15:32. doi: 10.1186/s12917-019-1773-4 (PMC6339306; doi:10.1186/s12917-019-1773-4)
Supplement: Supplementary file 2 — Figure S2. Optimization of the size of gold nanoparticles. Each point was photographed with two copies. (DOC 112 kb) [file 12917_2019_1773_MOESM2_ESM.doc]

The optimization of the size of gold nanoparticles

The optimization of gold nanoparticles was performed by using 16 nm, 24 nm, 30 nm and 40 nm gold nanoparticles to make the sandwich ICA. 80 μl sample solution (the concentration of PEDV was 5 μg/ml) was added to the test strip and photos were taken after reaction for 15 min.

**Results**


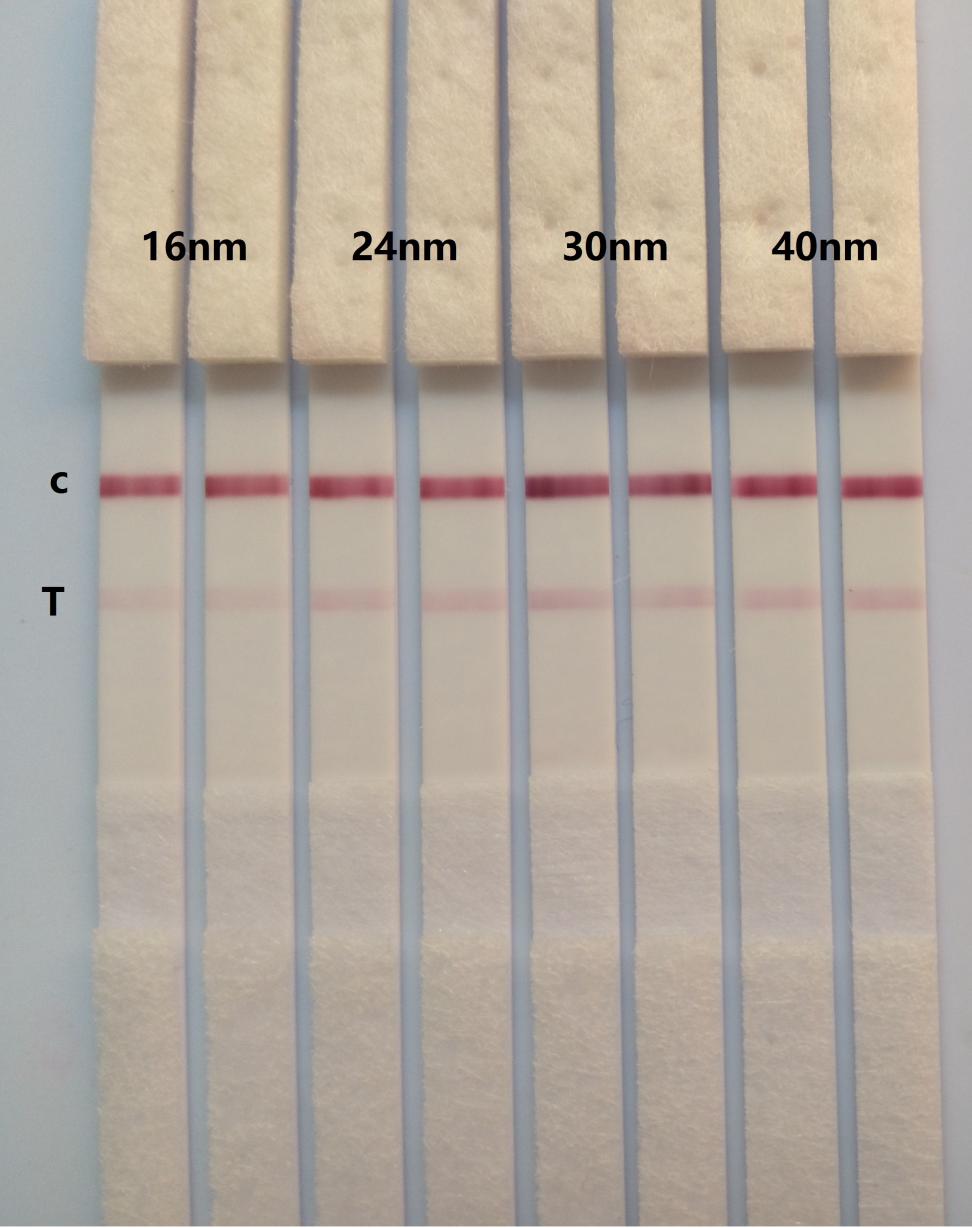


Fig. S2 Optimization of the size of gold nanoparticles. Each point was photographed with two copies.
