# Supplementary material for: A new immunochromatographic assay for on-site detection of porcine epidemic diarrhea virus based on monoclonal antibodies prepared by using cell surface fluorescence immunosorbent assay
Source: BMC Vet Res. 2019 Jan 18;15:32. doi: 10.1186/s12917-019-1773-4 (PMC6339306; doi:10.1186/s12917-019-1773-4)
Supplement: Supplementary file 3 — Figure S3. Optimization of the type of sample pad. (DOC 376 kb) [file 12917_2019_1773_MOESM3_ESM.doc]

The optimization of the type of sample pad

To optimize the type of sample pad, 5 kinds of sample pads (SB-06, G-2, G-8, GL-B04, and polyester film) were used to make the sandwich ICA. 80 μl PB (0.2 M，pH 7.4, containing 1% (w/v) Tween-20) was added to the test strip and photos were taken after reaction for 15 min.

**Results**


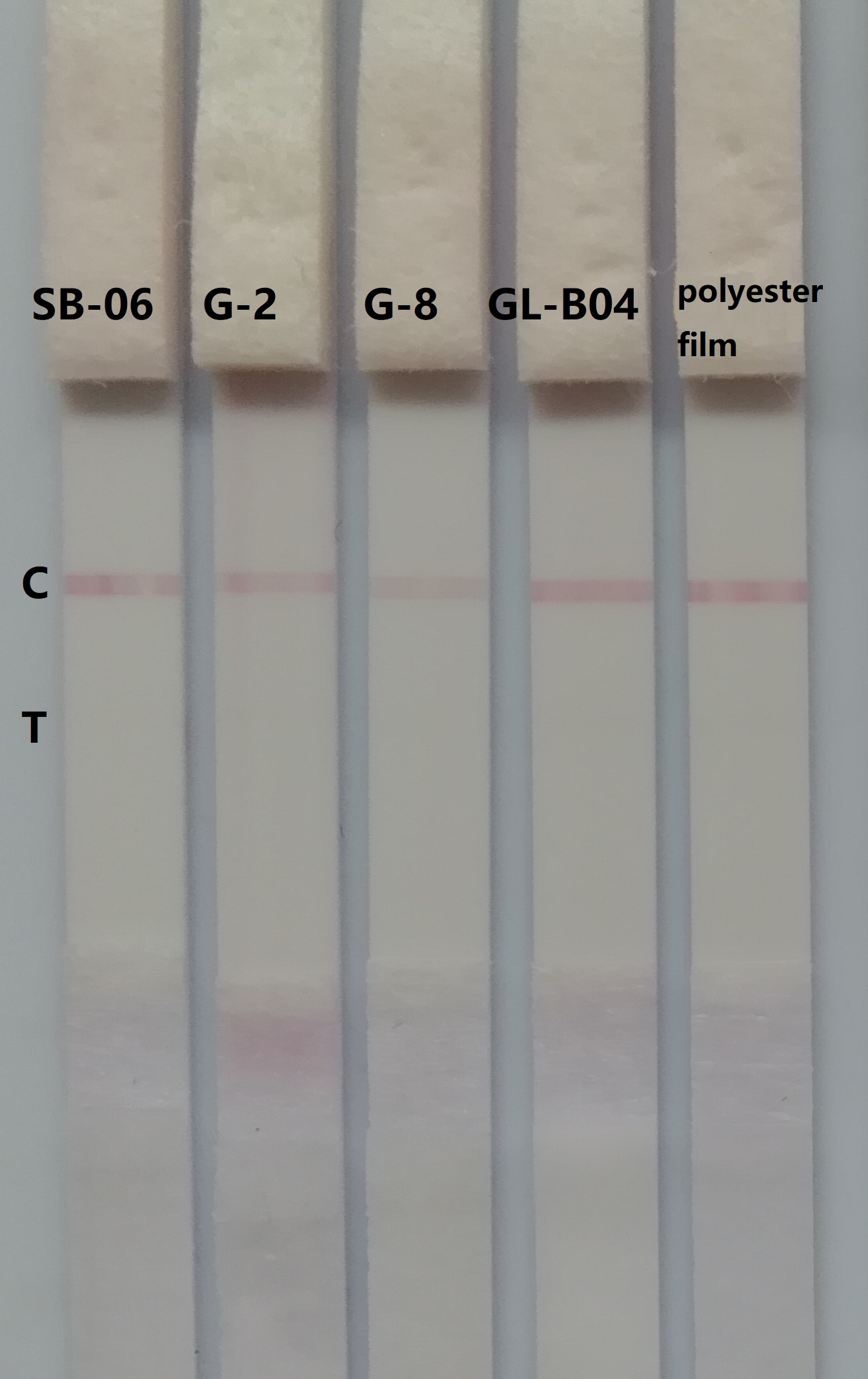


Fig. S3 Optimization of the type of sample pad.
