# Supplementary material for: A new immunochromatographic assay for on-site detection of porcine epidemic diarrhea virus based on monoclonal antibodies prepared by using cell surface fluorescence immunosorbent assay
Source: BMC Vet Res. 2019 Jan 18;15:32. doi: 10.1186/s12917-019-1773-4 (PMC6339306; doi:10.1186/s12917-019-1773-4)
Supplement: Supplementary file 6 — Figure S6. Optimization of the type of absorbent pad. Each point was photographed with two copies. (DOC 328 kb) [file 12917_2019_1773_MOESM6_ESM.doc]

The optimization of the type of absorbent pad

To optimize the type of absorbent pad, 4 kinds of absorbent pads (H2, H6, H7, and H8) were used to make the sandwich ICA. 80 μl sample solution (the concentration of PEDV was 5 μg/ml) was added to the test strip and photos were taken after reaction for 15 min.

**Results**


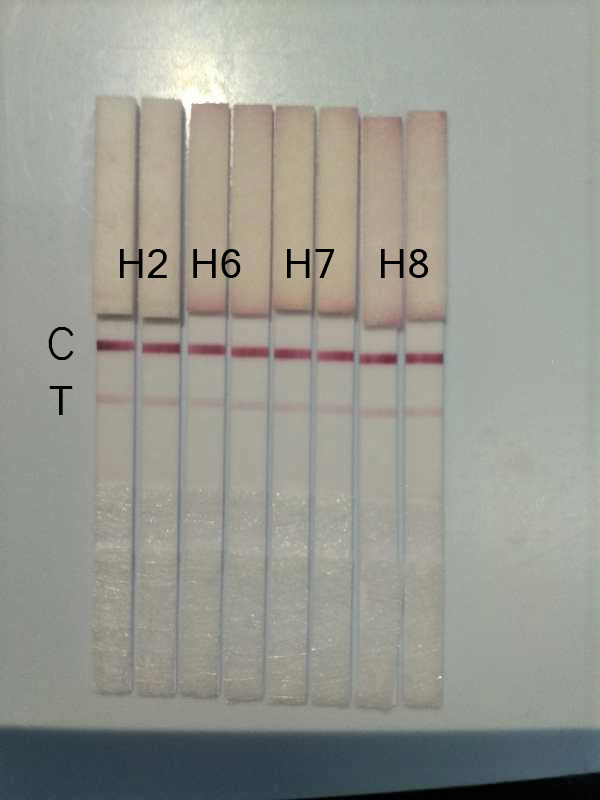


Fig. S6 Optimization of the type of absorbent pad. Each point was photographed with two copies.
