# Supplementary material for: A new immunochromatographic assay for on-site detection of porcine epidemic diarrhea virus based on monoclonal antibodies prepared by using cell surface fluorescence immunosorbent assay
Source: BMC Vet Res. 2019 Jan 18;15:32. doi: 10.1186/s12917-019-1773-4 (PMC6339306; doi:10.1186/s12917-019-1773-4)
Supplement: Supplementary file 7 — Figure S7. Optimization of the amount of tween-20 addition. Each point was photographed with two copies. (DOC 609 kb) [file 12917_2019_1773_MOESM7_ESM.doc]

The optimization of the amount of tween-20 addition

The optimization of the amount of tween-20 addition was performed by changing the amount of tween-20 (5, 10, 15, 20, or 40 μl) in sample solution. 80 μl sample solution (the concentration of PEDV was 40 μg/ml) was added to the test strip and photos were taken after reaction for 15 min.

**Results**


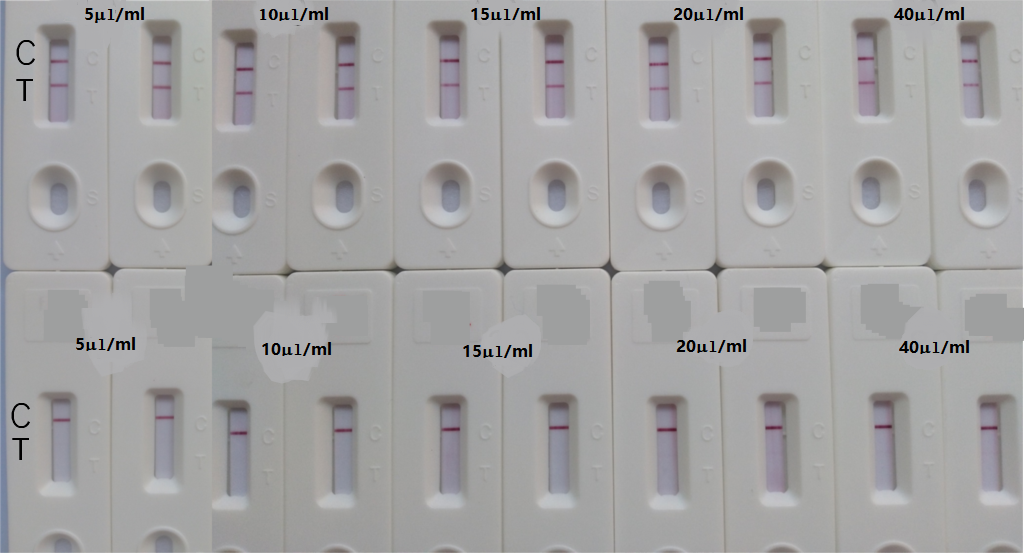


Fig. S7 Optimization of the amount of tween-20 addition. Each point was photographed with two copies.
