# Supplementary material for: A new immunochromatographic assay for on-site detection of porcine epidemic diarrhea virus based on monoclonal antibodies prepared by using cell surface fluorescence immunosorbent assay
Source: BMC Vet Res. 2019 Jan 18;15:32. doi: 10.1186/s12917-019-1773-4 (PMC6339306; doi:10.1186/s12917-019-1773-4)
Supplement: Supplementary file 8 — Figure S8. Optimization of the spray volume of AuNPs-mAb. (DOC 124 kb) [file 12917_2019_1773_MOESM8_ESM.doc]

The optimization of the spray volume of AuNPs-mAb

To optimize the spray volume of AuNPs-mAb, the conjugate pad with different spray volumes of AuNPs-mAb (3, 4, 5, 6, and 7 μl/cm) was used to make the sandwich ICA. 80 μl sample solution (the concentration of PEDV was 40 μg/ml) was added to the test strip and photos were taken after reaction for 15 min.

**Results**


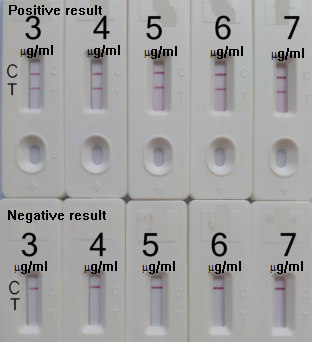


Fig. S8 Optimization of the spray volume of AuNPs-mAb
