# Supplementary material for: A new immunochromatographic assay for on-site detection of porcine epidemic diarrhea virus based on monoclonal antibodies prepared by using cell surface fluorescence immunosorbent assay
Source: BMC Vet Res. 2019 Jan 18;15:32. doi: 10.1186/s12917-019-1773-4 (PMC6339306; doi:10.1186/s12917-019-1773-4)
Supplement: Supplementary file 9 — Figure S9. Stability of the sandwich ICA for PEDV detection. a There was red line on the positive test strip’s T line. b There was red line on the positive test strip’s T line when the same experiment was done with the same test strips made one month ago. (DOC 163 kb) [file 12917_2019_1773_MOESM9_ESM.doc]

Stability of the sandwich ICA for PEDV detection

The stability of the sandwich ICA was tested as follows: 10 μl PEDV (Virus titre was expressed as TCID_50_, 10^6^ TCID_50_/ml) was diluted in 110 μl PB (0.2 M，pH 7.4, containing 1% (w/v) Tween-20) to make the positive sample solution. The negative sample solution was made of 80 μl PB (0.2 M, pH 7.4, containing 1% (w/v) Tween-20). 80 μl of positive sample solution and negative sample solution were added to the sample holes of test strips respectively and the photos of these test strips were taken to reserve the results after reaction for 15 min. The same experiment was conducted one month later with the same PEDV antigen stored at 4°C and ICA device stored in a 23°C storage cabinet with a 50% humidity.

**Results**


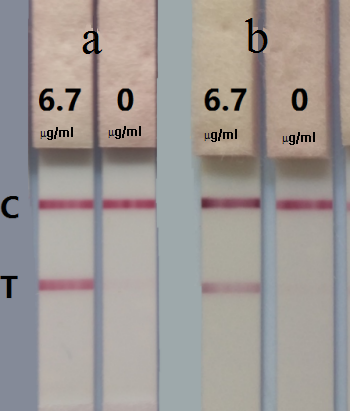


Fig. S9 Stability of the sandwich ICA for PEDV detection. a There was red line on the positive test strip’s T line. b There was red line on the positive test strip’s T line when the same experiment was done with the same test strips made one month ago.
